# Developing a Questionnaire to Assess Opinions Regarding the Importance of Bathing and Washing Without Water

**DOI:** 10.1093/geroni/igaa057.650

**Published:** 2020-12-16

**Authors:** Fabian Groven, Jan Hamers, Gaby Odekerken-Schröder, Sandra Zwakhalen

**Affiliations:** Maastricht University, Maastricht, Limburg, Netherlands

## Abstract

Bathing is one of the most performed activities among nurses. Although care recipients experience bathing as an important activity in daily living, nurses often undervalue this care task. We developed a questionnaire to measure nurses’ opinions regarding 1) the importance of the bed bath, and 2) a bathing innovation known as Washing Without Water. Construction of the questionnaire items was based on literature and interviews with nursing home residents (n=8), their family (n=5) and nurses (n=6). After items construction, nurses and nursing students (n=124) completed the questionnaire to assess the questionnaire’s internal consistency (IC) and construct validity. Cronbach’s alpha coefficients were analyzed as an indicator for IC and items were deleted if this resulted in improved IC. To analyze the construct validity, a Principal Component Analyses (PCA) with Direct Oblimin Factor rotation was performed. The final scale consists of two subscales. The first subscale measures nurses’ opinions about the importance of the bed bath and consists of 12 items. The second subscale consists of 17 items and aims to inventory nurses’ opinions about the Washing Without Water innovation. The Cronbach’s alpha coefficients are high (.81 for the first and .89 for the second subscale). The PCA results show a one factor loading for both subscales, explaining 33,20% and 37,08% of the variance for the first and second subscale respectively. Results indicate a reliable and valid questionnaire to measure nurses’ opinions related to the bed bath, which can support health care institutions in evaluating the bed bathing process.

